# Periodontal Disease in the Brazilian Population: A Retrospective Analysis on the 2013 National Health Survey to Identifying Risk Profiles

**DOI:** 10.1155/2022/5430473

**Published:** 2022-10-07

**Authors:** Thiago Antônio Raulino do Nascimento, José Vilton Costa, Ricardo Oliveira Guerra

**Affiliations:** ^1^Federal Institute of Education Science and Technology of Rio Grande do Norte, Natal, Rio Grande do Norte, Brazil; ^2^Subcoordination of Sanitary Surveillance of State of Rio Grande do Norte, Natal, Rio Grande do Norte, Brazil; ^3^Federal University of Rio Grande do Norte, Natal, Rio Grande do Norte, Brazil

## Abstract

Periodontal disease (PD) is a global public health problem with prevalence varying according to social and economic contexts; however, few studies have investigated the distribution of PD worldwide. PD is the host response to an infection or progression of a clinical condition, and the identification of modifiable risk factors for adequate clinical management of patients should be a priority in health policies directed to vulnerable population groups. In this context, we investigated the characteristics and risk factors for PD using the Brazilian National Health Survey 2013 (PNS-2013). A cluster analysis using the interdependence technique was applied to explore data on the risk of periodontitis. The presence or absence of a risk factor was analyzed using five variables (ten categories), while ordinal regression assessed risk profiles based on sociodemographic aspects of the Brazilian population. Individuals were classified as low (26.33%), medium (23.34%), or high risk (50.32%) for PD. Age, educational level, ethnicity, and Brazilian regions (except the North region) were significantly associated with risk for PD in the adjusted final regression model. Individual and social contexts were factors related to the high risk of PD in the Brazilian population. Our results highlight the need for public policies on healthy habits to prevent systemic diseases affecting oral health.

## 1. Introduction

Periodontal disease (PD) is a chronic condition characterized by the destruction of tooth-supporting tissues (gums) and structures (cementum, periodontal ligament, and alveolar bone) [1]. PD is one of the most prevalent conditions worldwide, and it may vary according to social and economic contexts [2]. Demographic transition and population aging may also favor the increasing prevalence of PD, especially in developing countries [3].

There are a wide variety of risk factors for the etiology of PD, including the subgingival microorganism process, lifestyle habits such as tobacco smoking, absence of diabetes mellitus control, cardiovascular mechanisms (high concentrations of cholesterol in the process of atherosclerosis), drug-induced disorders (decreased salivary flow from antihypertensives, narcotic analgesics, sedatives, antihistamines, and prolongated use), obesity, and inadequate responses to stress behavior [4]. Other risk factors for PD can be categorized as modifiable (smoking, no diabetes mellitus control, cardiovascular disease, drug-induced disorders, stress, and obesity) and nonmodifiable (osteoporosis, hematologic disorders, host immune response, female hormonal changes, and pregnancy) [4]. Social factors are also considered as risk factors for PD, such as advanced age, sex, educational level, and ethnicity [5, 6]; furthermore, low socioeconomic status can also influence other social characteristics and indirectly affect the evolution of PD [7, 8].

Given that PD is identified as a host response to an infection and not only the progression of a clinical condition, recognizing modifiable risk factors for adequate clinical management of patients should be a priority in health policies directed at vulnerable populations [9]. Data on PD are scarce and present variable results among countries in Latin America [10]. As PD is associated with chronic diseases and Brazil is experiencing an accelerated population aging process that affects various socioeconomic profiles, the analysis of associated risk factors among populations is needed. Therefore, this study investigated the characteristics of PD using the Brazilian National Health Survey of 2013 (PNS-2013) database [11] associated with PD. The PNS2013 has important information about anthropometric measures such as waist circumference and biochemical analyzes, that were not included in the posterior PNS editions. Thus, our objectives were to create a set of variables built from variables available only in PNS 2013 where, through a cluster analysis, it was possible to identify profiles of people at risk of presenting PD.

## 2. Material and Methods

We used the PNS-2013 database provided by the Brazilian Institute of Geography and Statistics (IBGE) [11], and data were analyzed using RStudio scripts [12]. PNS-2013 was performed in three stages according to a cluster sampling method, consisting of census tracts, residences, and interviews with residents aged over 18 years. The sample is representative of the Brazilian population and contains data from all regions, main federation units, and metropolitan areas (i.e., 65, 1 million private households and 200, 6 million individuals).

To identify profiles of people at risk for PD, we created a statistical variable considering specific variables from the PNS-2013 database. The following variables were included: use of health services (Module J), lifestyle (Module P), oral health (Module U), and laboratory information (Module W) (Table1). The selection of key variables was based on epidemiological evidence that reported modifiable risk factors for PD and the impacts on disease susceptibility [13–15]. Individuals with edentulism (9,379) were excluded from the study, resulting in a final sample of 42,728 individuals.

A cluster analysis using the interdependence technique was applied to explore data on the risk for PD and find an underlying structure in the set of key variables analyzed. This analysis was performed using a clustering algorithm or association between variables. Selected variables were recategorized to obtain a two-dimensional perceptual map: J013 (dental visit in the last 12 months, yes or no), P050 (smoker or nonsmoker), Q030 (diagnosis of diabetes, yes or no), U00203 (dental flossing, yes or no), and W00303 (waist circumference, risk or no risk). These variables (10 categories) were analyzed for the presence or absence of a risk factor. The following risk factors were considered: not visiting a dentist (J013); being a smoker, even if not daily (P050); a diagnosis of diabetes, except during pregnancy (Q030); and a circumference >94 cm for males and 80 cm for females (W00303).

Distances between points were used in a cluster analysis to obtain a dendrogram. Similarity level was defined using a clustering algorithm considering Hamming distance. After creating variables and categories, an ordinal regression assessed risk profiles based on the sociodemographic aspects of the Brazilian population (Table 2).

## 3. Results

Individuals were classified as low (26.33%), medium (23.34%), and high-risk (50.32%) for PD. Sociodemographic characteristics (male sex, advanced age, low educational level, and black ethnicity) were more prevalent in the high-risk PD group. Regarding lifestyle, smokers and those who did not use dental floss were more frequent. Regarding dental care, most individuals who did not visit a dentist and had an unsatisfactory perception of oral health were classified as high-risk (Table 3).

Table 4 presents the results of bivariate analysis before ordinal regression to verify the occurrence of high risk according to independent variables included in the model. Categories of each variable with a high-risk outcome were used as reference groups for ordinal regression.

Age, education level, ethnicity, and country regions (except the North) were significantly associated with the risk for PD in the adjusted final regression model (Table 5).

## 4. Discussion

We aimed to investigate PD characteristics in a sample of the Brazilian population to identify associated risk factors. Our results have an impact and open opportunities to discuss strategies to prevent and treat the consequences of PD. The high number of individuals at high risk for PD in our study is consistent with the Brazilian Survey of Buccal Health [16]. The heterogeneous prevalence of PD in Brazil may be explained by social inequalities observed in unfavorable economic areas (e.g., the North and Northeast regions), whereas contextual variables, such as income inequality, were recognized as a strong factor associated with severe PD [17].

In our study, the low educational level and ethnicity were social aspects associated with PD, corroborated by the literature in developing countries [10, 18, 19]. The use of dental care by disadvantaged people is an important challenge for public and universal health systems, since individuals with social inequalities (e.g., low educational level and income) [20]. We did not observe associations between sex and PD in our final regression model. Other studies demonstrated that males presented a high risk for PD in Brazil [17]. Hygiene habits and regular visits to the dentist may explain this difference since females are more likely to use health and dental services in Brazil [21].

Regarding modifiable and individual risk factors for PD, our cluster analysis indicated a high risk for metabolic conditions, such as obesity. The association between metabolic diseases and the worsening of PD is well described in the literature [22, 23]. Individuals with metabolic syndrome are 38% more likely to have PD than individuals without this condition [24].

The potential limitations of our study were related to the PNS design since causality cannot be inferred in ecological studies. Another limitation was the lack of clinical data regarding PD in the database. However, the identification of risk factors for PD is essential to public health. Data from PNS are easy to collect and may be used continuously in new editions of Brazilian household surveys to monitor disease and modifiable risk factors for PD that are common to other general health problems.

## 5. Conclusion

We identified a high-risk profile for PD among subjects who had factors such as diabetes, obesity, smoking, low adherence or access to dental visits, and a lack of dental flossing. The social context of individuals was also related to a high risk for PD, according to age, educational level, and sociodemographic characteristics. The identified risk profile highlights the need for public policies related to healthy habits to prevent systemic diseases that affect oral health and to avoid diseases such as PD that are preventable but often neglected.

## Figures and Tables

**Table 1:** PNS-2013 variables were used to construct a “risk profile for periodontal disease.” Source: PNS-2013.

PNS code variables	Description	Categories or values
J013	When did you last visit a dentist?	1. In the last 12 months
		2. From one- to less than two-years
		3. From two- to less than three-years
		4. Three or more years
		5. Never visited a dentist.

P050	Do you currently smoke tobacco products?	1. Yes, daily
		2. Yes, less than daily
		3. I currently do not smoke.

Q030	Has your doctor ever diagnosed you as diabetic?	1. Yes.
		2. Only during pregnancy
		3. No

U00203	Do you use dental floss?	1. Yes.
		2. No

W00303	Waist circumference (cm)	20 to 210

**Table 2:** Variables used in ordinal regression.

Variable	Description	Categorization
*Dependent variable*		
Periodontal risk	Construct (risk for periodontal disease)	1. Low risk 2. Medium risk 3. High risk

*Sociodemographic characteristics*		
C006	Sex	0. Female
		1. Male

C008	Age (years)	1. 18 to 24
		2. 25 to 39
		3. 40 to 59
		4. More than 60

C009	Ethnicity	1. White
		2. Brown
		3. Others
		4. Black

VDD004	Educational level	1. Middle school degree or incomplete high school degree
		2. High school degree or incomplete higher education degree
		3. Higher education degree
		4. Up to incomplete middle school degree

V001	Country regions	1. North
		2. Southeast
		3. South
		4. Midwest
		5. Northeast

**Table 3:** Analysis by risk categories for periodontal disease.

Variable	Periodontal risk level			
	Low	Medium	High	Total
	*n* (%)	*n* (%)	*n* (%)	*n* (%)
Sex				
Male	5134 (28.31)	3315 (18.8)	9683 (53.40)	18132 (42.44)
Female	6118 (24.87)	6658 (27.07)	11820 (48.06)	24596 (57.56)

Age (years)				
18 to 24	3056 (49.91)	794 (12.97)	2273 (37.12)	6123 (14.33)
25 to 39	5383 (30.50)	4099 (23.22)	8170 (46.28)	17652 (41.31)
40 to 59	2366 (16.28)	3999 (27.51)	8171 (56.71)	14536 (34.02)
Over 60	447 (10.12)	1081 (24.47)	2889 (65.41)	4417 (10.34)
Level of education				
Incomplete middle school degree	2058 (16.29)	1589 (12.66)	8975 (71.05)	12632 (29.56)
Middle school degree	1878 (27.61)	1256 (18.47)	3667 (53.92)	6801 (15.92)
High school degree	5132 (31.71)	4269 (26.37)	6785 (41.92)	16186 (37.88)
College degree	2184 (30.72)	2849 (40.08)	2076 (29.20)	7109 (16.64)
Ethnicity				
Black	955 (23.88)	746 (18.65)	2299 (57.48)	4000 (9.39)
White	4823 (27.45)	5102 (29.03)	7648 (43.52)	17573 (41.13)
Brown	5273 (25.74)	3964 (19.35)	11251 (54.92)	20488 (47.95)
Others	201 (30.13)	161 (24.14)	305 (45.73)	667 (1.56)
Regions				
Northeast	3092 (23.94)	2360 (18.27)	7465 (57.79)	12917 (30.23)
Southeast	2932 (27.62)	2958 (27.86)	4727 (44.52)	10617 (24.85)
South	1476 (27.54)	1654 (30.86)	2230 (41.60)	5360 (12.54)
Midwest	1500 (27.70)	1490 (27.52)	2425 (44.78)	5415 (12.67)
North	2252 (26.75)	1511 (17.95)	4656 (55.30)	8419 (19.70)
Self-perception of oral health				
Satisfactory	8565 (29.27)	7862 (26.86)	12840 (43.87)	29267 (68.50)
Unsatisfactory	2687 (19.96)	2111 (15.68)	8663 (64.36)	13461 (31.50)
Tobacco				
Nonsmoker	10013 (89.0)	9083 (91.1)	18324 (85.2)	37420 (87.58)
Smoker	1239 (11.0)	890 (8.9)	3179 (14.8)	5308 (12.42)
Odontology care				
Visit the dentist	7514 (66.8)	9973 (100.0)	3869 (18.0)	21356 (49.98)
Do not visit to the dentist	3738 (33.2)	0 (0.0)	17634 (82.0)	21372 (50.02)
Buccal care				
Use dental floss	9210 (81.9)	9973 (100.0)	6925 (32.2)	26108 (61.10)
No use dental floss	2042 (18.1)	0 (0.0)	14578 (67.8)	16620 (38.90)
Obesity				
Not obese	11252 (81.8)	0 (0.0)	3913 (18.2)	15165 (35.49)
Obese	0 (0.0)	9973 (100.0)	17590 (81.8)	27563 (64.51)
Diabetes				
Nondiabetic	11080 (98.5)	9496 (95.2)	20165 (93.8)	40741 (95.35)
Diabetic	172 (1.5)	477 (4.8)	1338 (6.2)	1987 (4.65)

**Table 4:** Bivariate analysis between periodontal disease frequency and sociodemographic and individual characteristics.

Variable	Periodontal risk level				Crude OR	IC (95%)		*P*-value
	Low	Medium	High	Total		Lower	Up	
	*n* (%)	*n* (%)	*n* (%)	*n* (%)				
Sex								
Male	5134 (28.31)	3315 (18.28)	9683 (53.40)	18132 (42.44)	—	—	—	0.001
Female	6118 (24.87)	6658 (27.07)	11820 (48.06)	24596 (57.56)	0.45	0.89	0.96	

Age (years)								
18 to 24	3056 (49.91)	794 (12.97)	2273 (37.12)		0.21	0.2	0.23	0.001
25 to 39	5383 (30.50)	4099 (23.22)	8170 (46.28)		0.41	0.38	0.44	0.001
40 to 59	2366 (16.28)	3999 (27.51)	8171 (56.71)		0.68	0.64	0.73	0.001
Over 60	447 (10.12)	1081 (24.47)	2889 (65.41)		—	—	—	

Level education								
Incomplete middle school degree	2058 (16.29)	1589 (12.66)	8975 (71.05)		—	—	—	—
Middle school degree	1878 (27.61)	1256 (18.47)	3667 (53.92)		0.46	0.44	0.49	0.001
High school degree	5132 (31.71)	4269 (26.37)	6785 (41.92)		0.31	0.3	0.33	0.001
College degree	2184 (30.72)	2849 (40.08)	2076 (29.20)		0.24	0.23	0.26	0.001

Ethnicity								
Black	955 (23.88)	746 (18.65)	2299 (57.48)	4000 (9.39)	—	—	—	—
White	4823 (27.45)	5102 (29.03)	7648 (43.52)	17573 (41.13)	0.65	0.61	0.69	0.001
Brown	5273 (25.74)	3964 (19.35)	11251 (54.92)	20488 (47.95)	0.9	0.84	0.96	0.001
Others	201 (30.13)	161 (24.14)	305 (45.73)	667 (1.56)	0.65	0.56	0.76	0.001

Regions								
Northeast	3092 (23.94)	2360 (18.27)	7465 (57.79)	12917 (30.23)	—	—	—	—
Southeast	2932 (27.62)	2958 (27.86)	4727 (44.52)	10617 (24.85)	0.66	0.63	0.69	0.001
South	1476 (27.54)	1654 (30.86)	2230 (41.60)	5360 (12.54)	0.61	0.58	0.65	0.001
Midwest	1500 (27.70)	1490 (27.52)	2425 (44.78)	5415 (12.67)	0.66	0.62	0.7	0.001
North	2252 (26.75)	1511 (17.95)	4656 (55.30)	8419 (19.70)	0.89	0.84	0.93	0.001

Self-perception of oral health								
Satisfactory	8565 (29.27)	7862 (26.86)	12840 (43.87)	29267 (68.50)	0.47	0.45	0.49	0.001
Unsatisfactory	2687 (19.96)	2111 (15.68)	8663 (64.36)	13461 (31.50)	—	—	—	—

**Table 5:** . The ordinal logistic final model of individuals with susceptibility to periodontal disease, according to sex, age group, ethnicity, level of education, country region, and self-perception of oral health.

Variable	Periodontal risk level				Adjusted OR	IC (95%)		*P*-value
	Low	Medium	High	Total		Lower	Up	
	*n* (%)	*n* (%)	*n* (%)	*n* (%)				
Sex								
Male	5134 (28.31)	3315 (18.28)	9683 (53.40)	18132 (42.44)	—	—	—	
Female	6118 (24.87)	6658 (27.07)	11820 (48.06)	24596 (57.56)	1.011	0.974	1.05	0.563

Age (years)								
18 to 24	3056 (49.91)	794 (12.97)	2273 (37.12)		0.219	0.202	0.238	0.001
25 to 39	5383 (30.50)	4099 (23.22)	8170 (46.28)		0.45	0.42	0.483	0.001
40 to 59	2366 (16.28)	3999 (27.51)	8171 (56.71)		0.713	0.665	0.765	0.001
Over 60	447 (10.12)	1081 (24.47)	2889 (65.41)		—	—	—	

Level education								
Incomplete middle school degree	2058 (16.29)	1589 (12.66)	8975 (71.05)		—	—	—	
Middle school degree	1878 (27.61)	1256 (18.47)	3667 (53.92)		0.618	0.581	0.658	0.001
High school degree	5132 (31.71)	4269 (26.37)	6785 (41.92)		0.429	0.408	0.451	0.001
College degree	2184 (30.72)	2849 (40.08)	2076 (29.20)		0.298	0.281	0.316	0.001

Ethnicity								
Black	955 (23.88)	746 (18.65)	2299 (57.48)	4000 (9.39)	—	—	—	
White	4823 (27.45)	5102 (29.03)	7648 (43.52)	17573 (41.13)	0.817	0.762	0.877	0.001
Brown	5273 (25.74)	3964 (19.35)	11251 (54.92)	20488 (47.95)	0.914	0.853	0.979	0.012
Others	201 (30.13)	161 (24.14)	305 (45.73)	667 (1.56)	0.711	0.606	0.834	0.001

Regions								
Northeast	3092 (23.94)	2360 (18.27)	7465 (57.79)	12917 (30.23)	—	—	—	
Southeast	2932 (27.62)	2958 (27.86)	4727 (44.52)	10617 (24.85)	0.732	0.695	0.772	0.001
South	1476 (27.54)	1654 (30.86)	2230 (41.60)	5360 (12.54)	0.722	0.676	0.771	0.001
Midwest	1500 (27.70)	1490 (27.52)	2425 (44.78)	5415 (12.67)	0.743	0.698	0.791	0.001
North	2252 (26.75)	1511 (17.95)	4656 (55.30)	8419 (19.70)	0.999	0.945	1.057	0.983

Self-perception of oral health								
Satisfactory	8565 (29.27)	7862 (26.86)	12840 (43.87)	29267 (68.50)	0.631	0.604	0.659	0.001
Unsatisfactory	2687 (19.96)	2111 (15.68)	8663 (64.36)	13461 (31.50)	—	—	—	

## Data Availability

Data from the 2013 National Health Survey (PNS 2013) used to support the findings of this study (PERIODONTAL DISEASE AND ASSOCIATED FACTORS IN THE BRAZILIAN POPULATION) were provided by Instituto Brasileiro de Geografia e Estatística (IBGE) in a public domain repository. Access to this data can be made through the link:https://www.ibge.gov.br/estatisticas/sociais/saude/9160-pesquisa-nacional-de-saude.html?=∼∼∼∼∼∼∼∼∼^∼^∼^∼^∼∼∼∼∼∼∼∼∼∼∼amp;t=microdados, to access the microdata and variable dictionary. Analysis was also made available by IBGE using the *R* software. We used this path in our research, https://cran.r-project.org/web/packages/PNSIBGE/index.html.
